# How genetic analysis may contribute to the understanding of avoidant/restrictive food intake disorder (ARFID)

**DOI:** 10.1186/s40337-022-00578-x

**Published:** 2022-04-15

**Authors:** Hannah L. Kennedy, Lisa Dinkler, Martin A. Kennedy, Cynthia M. Bulik, Jennifer Jordan

**Affiliations:** 1 Department of Psychological Medicine, University of Otago, Christchurch, PO Box 4345, Christchurch, 8140 New Zealand; 2 Department of Medical Epidemiology and Biostatistics, Karolinska Institutet, PO Box 281, 171 77 Stockholm, Sweden; 3 Gillberg Neuropsychiatry Centre, Sahlgrenska Academy, University of Gothenburg, 411 19 Gothenburg, Sweden; 4 Department of Pathology and Biomedical Science, University of Otago, Christchurch, New Zealand; 5 Department of Psychiatry, University of North Carolina at Chapel Hill, CB #7160, 101 Manning Drive, Chapel Hill, NC 27599-7160 USA; 6 Department of Nutrition, University of North Carolina at Chapel Hill, Chapel Hill, NC 27599 USA

**Keywords:** GWAS, Heritability, Psychiatric genetics, Fussy eating

## Abstract

Avoidant/restrictive food intake disorder (ARFID) was introduced in the fifth edition of the Diagnostic and Statistical Manual of Mental Disorders (DSM-5). Unlike anorexia nervosa, ARFID is characterised by avoidant or restricted food intake that is not driven by weight or body shape-related concerns. As with other eating disorders, it is expected that ARFID will have a significant genetic risk component; however, sufficiently large-scale genetic investigations are yet to be performed in this group of patients. This narrative review considers the current literature on the diagnosis, presentation, and course of ARFID, including evidence for different presentations, and identifies fundamental questions about how ARFID might fit into the fluid landscape of other eating and mental disorders. In the absence of large ARFID GWAS, we consider genetic research on related conditions to point to possible features or mechanisms relevant to future ARFID investigations, and discuss the theoretical and clinical implications an ARFID GWAS. An argument for a collaborative approach to recruit ARFID participants for genome-wide association study is presented, as understanding the underlying genomic architecture of ARFID will be a key step in clarifying the biological mechanisms involved, and the development of interventions and treatments for this serious, and often debilitating disorder.

## Plain English summary

Avoidant/restrictive food intake disorder (ARFID) can be a severe and debilitating eating disorder, where individuals limit food intake for reasons unrelated to the weight and body image concerns observed in anorexia nervosa. Although genetics is known to play a significant role in other eating disorders such as anorexia nervosa and bulimia nervosa, only one study has investigated the genetic background of ARFID, and this was limited to those with ARFID within an autism cohort. This narrative review describes current knowledge about the clinical characteristics of ARFID and highlights current knowledge gaps, setting the scene for a discussion of how existing research findings about the genetics of related conditions might help guide genetic research about ARFID. A large genome-wide association study (GWAS) is recommended as the first step to addressing some of the fundamental biological questions around ARFID and will lay the framework for development of interventions and treatments that target ARFID at a biological level.

## Introduction

Avoidant/restrictive food intake disorder (ARFID) is a potentially severe and debilitating eating disorder, where individuals limit food intake for reasons unrelated to the weight and body image concerns observed in anorexia nervosa. As it has only been included as an eating disorder in DSM-5, it is relatively under-researched and there is much that is unknown about this condition. This narrative review briefly summarises pertinent literature on ARFID, in the context of considering it to be a complex disorder with likely but yet unclear genetic underpinnings, and highlights knowledge gaps and methodological considerations. It then considers how genetic research is well positioned to address some of these issues, including a discussion of the genetic findings from related conditions, the impact of genetic research on our conceptualisation of ARFID, and proposes an established framework of consortium science to advance the field.

ARFID is defined as an eating or feeding disturbance, manifested by persistent failure to meet appropriate nutritional and/or energy needs. Potential effects of not meeting these needs include significant weight loss or growth compromise, severe malnutrition, dependence on nutritional supplementation, and/or marked interference with psychosocial functioning [1]. The consequences of ARFID can be severe. Malnutrition from a restricted diet can, for instance, lead to serious vitamin deficiencies including vitamin D, C, and B9, contributing to osteoporosis, scurvy, and myelodysplasia respectively [1–3]. Consuming less nutrients than the body requires can lead to severe cardiovascular, gastrointestinal, neurological, and endocrine changes [4]. ARFID also adversely affects psychosocial functioning. Individuals may find social eating difficult due to distress around food, or embarrassment at their restricted eating behaviour. Children often experience high levels of stress and conflict with parents around mealtimes. Communal eating at home, school, or in the workplace are common situations that become difficult to navigate.

Avoidant or restrictive eating behaviour in infants and young children has been previously described using terms such as fussy/picky eating, infantile anorexia, feeding disorder, and food avoidance emotional disorder. Although these terms have been inconsistently defined, and not all behaviour described using these terms will be relevant to ARFID (i.e., oral-motor dysfunction causing a feeding disorder), literature in the area of paediatric feeding/eating difficulties represents an important foundation that may help inform the pathology of ARFID.

The exact prevalence of ARFID in the general population is unknown, but likely lies in the range of 0.5–5%, both in children [5–8] and adults [9–12]. Prevalence estimates from clinical eating disorder (ED) populations range from 1.5 to 64% [13–20].

In contrast to most other EDs, males seem to be almost as frequently affected by ARFID as females [5, 6, 11, 12]. In studies from child and adolescent ED programs, males account for 20–35% of ARFID cases [14, 16, 17, 19–21], while 35–68% of children in paediatric feeding disorder programs are male (median age: 2–4 years). Onset of ARFID can occur at any age [22, 23], although the current literature is predominantly on childhood or adolescent presentation [24–26].

### ARFID presentation

The aetiology of other eating disorders is now accepted to be a dynamic and complex interplay between the genetic makeup of an individual and a variety of environmental factors, including the possibility of gene-environment interactions [27]. ARFID displays moderate phenotypic overlap with other eating disorders, particularly the restricting behaviour, low weight and malnutrition seen in AN. Preliminary evidence for diagnostic transition from ARFID to AN [21, 28, 29] also suggests shared aetiology. Furthermore, there is considerable phenotypic overlap, and comorbidity, between ARFID and neurodevelopmental disorders such as ASD or ADHD [15, 16, 30], and other psychiatric conditions including anxiety and OCD [14, 15]. This is in contrast to AN, for example, where comorbid neurodevelopmental disorders are less frequent, and depression is more prevalent than anxiety [14, 15, 30–32].

DSM-5 recognises significant variability in presentation of ARFID, and provides three examples: (1) an apparent lack of interest in eating; (2) avoidance based on the sensory characteristics of food; and (3) a concern about the aversive consequences of eating. These presentations are not mutually exclusive, but represent three distinct mechanisms of food restriction or avoidance observed in ARFID. Although these proposed presentations are not officially recognised in classification systems, they are commonly used in the literature and we have referred to them as ‘limited intake’, ‘limited variety’, and ‘aversive’ presentations in the following text. Although individual case reports demonstrate each of the three proposed presentations, a mixed clinical presentation is often observed in ARFID patients [30, 33] and Thomas et al. [34] propose that individuals with ARFID can present with any degree of features.

The limited intake presentation includes cases previously described with terms such as food avoidance emotional disorder, infantile anorexia, or restrictive eating. Patients may present with a low appetite, a lack of interest in eating, or behaviours that reduce intake such as taking small bites, and excessive chewing and slow eating [35]. Individuals tend to have a lower average BMI than other ARFID presentations [13, 33, 35] and malnutrition in children with this presentation is associated with increasing psychopathology up to the fragile pre-pubescent period, a critical period for onset of other eating disorders (EDs) [36]. Persistent under-eating in children predicts adolescent anorexia nervosa (AN) [37], raising the possibility that ARFID (particularly the limited-intake presentation) could be a risk factor for development of other EDs later in life or potentially even a prodromal presentation.

The limited variety presentation can be initially mistaken for selective or ‘picky’ eating, which is common in early childhood [38, 39]. Even where this selective eating reaches the threshold for ARFID diagnosis, individuals tend not to be underweight [13] but instead they may suffer from micronutrient malnutrition [15]. Mechanisms that tend to limit variety of food intake include, but are not limited to, neophobia (fear of the unfamiliar) and sensory aversion. Studies on selective eating indicate a high degree of comorbid anxiety, autism spectrum disorder (ASD), obsessionality/rigidity, and sensory sensitivity [33, 40, 41]. The limited variety presentation includes a higher prevalence of affected boys compared with other restrictive eating disorders [42], which may be due in part to comorbid neurodevelopmental disorders that disproportionately affect males [43–45].

An aversive outcome ARFID presentation is characterised by food avoidance or restriction based on a fear of undesired consequences such as choking, allergic reaction, nausea or vomiting, illness etc. For many, this occurs acutely following exposure to a traumatic event, and individuals frequently display a concurrent anxiety disorder [13, 30, 35]. Patients with this presentation are reported to have a shorter length of illness and are more frequently hospitalised (associated with more acute weight loss and associated health concerns) than the other two presentations [13, 30, 33, 35].

### Comorbidity

The current literature on ARFID highlights a high degree of comorbidity with other psychiatric, neurodevelopmental, and medical conditions, with approximately 50% of ARFID cases having a current comorbid diagnosis [40].

Generalized anxiety disorder, social anxiety disorder, and obsessive–compulsive disorder (OCD) are commonly reported to co-occur with ARFID. Compared to comorbidity patterns in other EDs, those with ARFID have relatively higher prevalence of anxiety disorders (35–73%), but lower prevalence of depression (15–35%) [14, 15, 30–32]. The latter may be an age dependent factor [46].

Individuals with autism spectrum disorder (ASD) often display rigid food preferences, relating to hypersensitivity to sensory elements such as texture and smell [47]. Similarly, the prevalence of feeding problems is higher among children with attention deficit hyperactivity disorder (ADHD) where barriers to eating include impulsivity, being easily bored or distracted, frequent talking, difficulty remaining seated at the table to complete a meal and reduced appetite due to prescribed stimulant medication [48]. Reports of ARFID and comorbid ADHD often demonstrate limited intake ARFID presentation features [13, 48].

In general, neurodevelopmental disorders might be more commonly present in ARFID than in AN [15, 16, 30]. In children and adolescents with ARFID, 3–23% are estimated to have comorbid ASD or ADHD, where 10–31% have learning difficulties/disorders, and 26–38% have intellectual disability or general developmental delay [15, 16, 30, 46, 49].

ARFID may also develop secondary to a history of medical problems such as gastrointestinal functional motility (e.g. gastroesophageal reflux disease [50]), malabsorption, inflammation (e.g. Crohn’s disease [23]), or food allergies [16]. Behavioural overlap also occurs between ARFID and paediatric acute-onset neuropsychiatric syndrome (PANS), including the PANS subgroup paediatric autoimmune neuropsychiatric disorder associated with streptococcal infections (PANDAS).

### Assessment and treatment of ARFID

Given the relatively recent introduction of ARFID as a diagnostic category, limited work has been conducted validating screening and diagnostic tools. In a recent review, Dinkler and Bryant–Waugh provide an overview of existing assessments of ARFID including their validation status [51].

No evidence-based treatment guidelines for ARFID exist, but Bryant-Waugh and Higgens [52] suggest that ARFID treatments should be based on evidence-based interventions implemented in other EDs. For example, an adapted cognitive behavioural therapy is being trialled in ARFID [53]. Pharmaceutical interventions in eating disorders are generally considered less effective than psychotherapy, and are usually implemented as an adjunct to other interventions, or as a second-line treatment. Currently, no approved medications for ARFID exist.

### ARFID genetics, what do we (not) know?

The roots of psychiatric genetics lie in family and twin studies that provide the first evidence that a disorder or condition aggregates in families, and can quantify the relative contribution of genetics, and shared or unique environment. No family or twin studies of ARFID have yet been published, and although there is one genetically-informed investigation of ARFID [54], this was performed with a sample of 3142 genotyped probands with ASD, of whom < 20% were classed as high-risk for ARFID. While ASD and ARFID frequently co-occur (as described above), far from all people with ARFID have ASD, limiting the generalizability of the results in this ASD group to the whole population with ARFID. The authors present a moderate, small nucleotide polymorphism (SNP)-based heritability of a continuous ARFID risk score, comparable to estimates in other EDs [55], but with relatively large confidence intervals (CIs; 0.45, 95% CI 0.13–0.76) [54]. Although the authors of this study acknowledge it was likely underpowered for gene discovery, one SNP on chromosome 5 did reach genome wide significance. The closest gene to this locus, *ZWIM6*, is a known neurodevelopmental gene that has been previously implicated in schizophrenia [56], and intellectual disability (ID) [57]. A de novo variant in *ZWIM6* has been found in unrelated cases of ID [58], with significant gastrointestinal symptoms (including gastroesophageal reflux disease), which are a common precursor for ARFID; this may warrant future investigation as a phenotype of interest.

In the absence of well-powered genetic studies on ARFID, we can also consider studies of similar or related traits as partial proxies for an ARFID phenotype. Related traits that have been studied using genetic epidemiology methods include appetite, rate of eating, food fussiness, phobic avoidance of food, food preferences, sensory reactivity, and nutrient intake. Current literature estimates moderate to high heritability for many specific eating behaviours which are implicated in ARFID (Table 1).

**Table 1:** Heritability measures (best fit model) of traits related to ARFID presentation. A summary of twin/family study derived heritability estimates

References	Age range	Study metrics	Specific behaviour or trait	Additive genetic variance (a^2^)	Non-additive genetic variance (d^2^)	Shared environmental variance (c^2^)	Non-shared environmental variance (e^2^)
Fildes et al. (2014) [107]	3.5 ± 0.27 y	Gemini Study: 1343 twin pairs, n = 458 [MZ], n = 872 [DZ], 50.4% female Instrument: 114 item parent-report questionnaire on food preferences	Vegetable preference	0.54 (0.47–0.63)	–	0.35 (0.27–0.42)	0.11 (0.10–0.13)
			Fruit preference	0.53 (0.45–0.61)	–	0.35 (0.26–0.43)	0.13 (0.11–0.15)
			Protein preference	0.48 (0.40–0.57)	–	0.37 (0.27–0.45)	0.15 (0.13–0.17)
			Dairy preference	0.54 (0.47–0.60)	–	0.54 (0.47–0.60)	0.19 (0.16–0.22)
			Starch preference	0.32 (0.26–0.38)	–	0.57 (0.51–0.62)	0.11 (0.10–0.13)
Breen et al. (2006) [108]	4–5 y	Twins Early Development Study (TEDS): 214 same-sex twin pairs, n = 103 [MZ] n = 111 [DZ], 52% female Instrument: Mother-report questionnaire on food preferences (95 food items)	Vegetable preference	0.37 (0.2–0.58)	–	0.51 (0.30–0.66)	0.13 (.09–.17)
			Dessert preference	0.2 (0.04–0.38)	–	0.64 (0.46–0.77)	0.16 (.12–.22)
			Meat and fish preference	0.78 (0.63–0.92)	–	0.12 (0.00–0.27)	0.10 (.08–.12)
			Fruit preference	0.51 (0.37–0.68)	–	0.32 (0.16–0.46)	0.17 (.14–.20)
Liu et al. (2013) [59]	11–13 y	University of Southern California (USC) Twin study: 358 twin pairs, n = 188 [MZ], n = 170 [DZ] Instrument: 3 day food diary	Fat intake	0.44 (0.28–0.58)	–	–	0.56 (0.42–0.72)
			Protein intake	0.31 (0.13–0.47)	–	–	0.69 (0.53–0.88)
			Carbohydrate intake	0.43 (0.25–0.58)	–	–	0.57 (0.42–0.75)
			Mineral intake	0.45 (0.29–0.59)	–	–	0.55 (0.41–0.71)
			Vitamin intake	0.21 (0.00–0.41)	–	0.04 (0.00–0.34)	0.75 (0.59–0.93)
Fildes et al. (2016) [63]	3.5 ± 0.3 y	Gemini Study: 1330 twin pairs, n = 458 [MZ], n = 872 [DZ], 50.5% female Instrument: 114 item parent report questionnaire on food preferences [107] + CEBQ [109]	Food fussiness	0.78 (0.73–82)	–	0.05 (0.02–0.09)	0.17 (0.15–0.2)
Smith et al. (2017) [64]	16 m	Gemini Study: 1932 twin pairs, n = 626 [MZ], n = 1306 [DZ], 50.6% female Instrument: Parent-report CEBQ	Food fussiness	0.46 (0.41–0.52)	–	0.46 (0.40–0.51)	0.09 (0.08–0.10)
			Food neophobia	0.58 (0.5–0.67)	–	0.22 (0.14–0.30)	0.19 (0.17–0.22)
Cooke et al. (2007) [65]	8–11 y	Twins Early Development Study (TEDS): 5390 twin pairs, n = 1913 [MZ], 3477 [DZ], 51.4% female Instrument: 4 item version of CFNS [110]	Food neophobia	0.78 (0.76–0.79)	–	–	0.22 (0.21–0.24)
Knaapila et al. (2007) [66]	Adult	Migraine family study—28 Finnish families: 105 females, 50 males Instrument: FNS [110] (10 item version) + FNS-R (6 item version of FNS)	Food neophobia	FNS 0.69*	–		0.31
				FNSR 0.66 *	–		0.34
		UK adult twin registry: 468 female twin pairs, n = 211 [MZ], n = 257 [DZ] Instrument: FNS [90] (10 item version) + FNS-R (6 item version of FNS)	Food neophobia	FNS 0.10 (0.00–0.56)	0.56 (0.09–0.73)	–	0.33 (0.27–0.41)
				FNSR 0.13 (0.00–0.59)	0.53 (0.06–0.72)	–	0.34 (0.28–0.41)
Llewellyn et al. (2010) [111]	Infant (~ 8 m)	Gemini Study: 2334 twin pairs, n = 729 [MZ], n = 1605 [DZ} Instrument: BEBQ (17 items)[112]	Rate of eating	0.84 (0.79–0.86)	–	0.00 (0.0–0.05)	0.16 (0.14–0.17)
			Satiety responsiveness	0.72 (0.65–0.80)	–	0.12 (0.05–0.19)	0.16 (0.14, 0.17)
			Feeding responsiveness	0.59 (0.52–0.65)	–	0.30 (0.24, 0.36)	0.11 (0.10, 0.13)
			Enjoyment of food	0.53 (0.43–0.63	–	0.45 (0.35, 0.54)	0.03 (0.02, 0.04)
Herle et al. (2017) [113]	5 y	Gemini Study: 1027 twin pairs, n = 346 [MZ], n = 681 [DZ]. Instrument: CEBQ	Emotional over eating	0.07 (0.06–0.09)	–	0.90 (0.89–0.92)	0.02 (0.02–0.03)
			Emotional under eating	0.07 (0.06–0.09)	–	0.91 (0.90–0.92)	0.02 (0.02–0.02)
Taylor et al. (2018) [114]	9–12 y	Child and Adolescent Twin Study in Sweden (CATSS): 12,419 twin pairs, n = 3586 [MZ], n = 8833 [DZ] Instrument: A-TAC Perception module (5 items) [115]	Sensory reactivity	Males 0.71 (0.68–0.74)	–	–	0.29 (0.26–0.32)
				Females 0.66 (0.61–0.69)	–	–	0.34 (0.31–0.39)

Variance estimates provided with 95% CI in parentheses

*MZ* monozygotic, *DZ* dizygotic, *BEBQ* Baby Eating Behaviour Questionnaire, *A-TAC* Autism-Tics, ADHD and other Comorbidities inventory, *CEBQ* Children’s Eating Behaviour Questionnaire, *CFNS* Child Food Neophobia Scale, *FNS* Food Neophobia Scale

^*^Familiarity estimate (a^2^ + c^2^), CI not presented

In a cohort of more than 1500 twins, the estimated heritability of food intake was 0.21–0.48, and the heritability of micronutrient intake was 0.21–0.45 [59]. Macronutrient and total energy intake have also been reported to be influenced by genetic factors (heritability estimated at 0.30–0.45) [59], with little contribution of shared environmental factors, particularly in adults [60, 61]. Highly heritable dietary preferences (such as for meat, fruit and vegetables, or carbohydrates) may implicate genes involved in taste perception as a possible contributor to food intake [62]. This is supported by strong phenotypic associations between toddler food fussiness and lower liking for nutritious foods such as vegetables and fruit in three year-old twins [63]. Twin and family studies additionally suggest high heritability of food neophobia in children (0.58–0.78) [64, 65], and moderate heritability in adults (0.66–0.69) [66].

Heritability of food fussiness estimates range from 0.46–0.78 [63, 64], and food preferences, particularly with regard to vegetables and fruit, may be partly mediated by an increased sensitivity to bitterness [63, 67]. Perceived bitterness of the compounds 6-n-propylthioruracil (PROP) and phenylthiocarbamide (PTC) has been attributed to genetic variants of the bitter gene receptor family member *TAS2R38*. Variation at this locus accounts for 55–85% of the variance in bitterness detection in response to PTC exposure [68]. This provides one example of heritable taste perception that may influence food fussiness, particularly in response to higher concentrations of bitter compounds found in cruciferous vegetables and citrus fruits like grapefruit.

Based on the findings of Koomar et al. [54] and the twin/family study data of related phenotypes above, it is reasonable to expect that the ARFID phenotype(s) will be at least moderately heritable, and that the degree of heritability and nature of the underlying genetic variants may differ across ARFID presentations.

### Psychiatric genetics strategies applicable to ARFID

It is clear that genetic influences on psychiatric conditions, including EDs, generally arise from a complex polygenic landscape, with potentially hundreds or thousands of low effect alleles [69]. Rare, highly penetrant variants, including copy number variants (CNVs) do not explain the majority of ED phenotypic variance. CNVs *are* implicated in neurodevelopmental [70, 71] and psychiatric disorders [72–74], and given the association with neurodevelopmental disorders may be more likely to occur in ARFID. However, it seems probable that common, small effect alleles will have a greater overall impact on phenotypic variance. Based on knowledge about the genetic landscape of other psychiatric conditions, and the expectation that genetic factors in ARFID may be similar, we consider below how best to approach an effective genetic analysis of ARFID.

#### ARFID genome-wide association study (GWAS) design

Genome-wide association studies (GWAS) are an essential tool to not only identify genetic loci associated with disease, but to provide a comprehensive dataset for subsequent analyses such as cross-disorder analyses, polygenic risk scores (PRS), and investigations into the functional impact of identified variants which may impact diagnosis, prognosis and treatment. The value of eating disorder GWAS to identify new associations between DNA variants and the traits they influence has been demonstrated in anorexia nervosa (AN) [75], where important metabolic and anthropometric associations encouraged reconceptualization of AN as a metabo-psychiatric disorder and identified an important direction for future research.

A significant challenge in GWAS is achieving sufficient statistical power given the generally small effect sizes of the loci sought, which requires genotyping and phenotyping of large numbers of cases and controls. A projection by Koomar et al. [54] suggests the number of participants required to achieve sufficient power for further ARFID gene discovery to be n = 10,000. Meta-analysis has proven a powerful tool for enhancing the power of individual GWAS discovery datasets, and identifying increasing numbers of genetic risk variants. However, meta-analysis involves the drawing together of multiple independent GWAS datasets, the development of which depends on funding and research priorities. This may well require a period of many years to achieve, although the long-term effort has proven extremely valuable for other conditions [56].

Because ARFID is often reported to be comorbid with one or more psychiatric or neurodevelopmental conditions, accurate phenotyping of cases will also be critical for success. Rich phenotyping will enable effective definition of subgroups or dominant presentations in downstream analyses, allowing the resolution of genetic factors that are specific to ARFID versus those that contribute to comorbid traits.

The type of control group used in genetic studies is important to consider, including for ARFID. When considering the sample sizes required, the use of unscreened controls (which can be simpler and cheaper to collect, but run the risk of case contamination) is a strategy that has been commonly employed [76, 77]. Increasing the number of control samples can compensate for this when using unscreened controls, as long as care is taken to correctly calculate the SNP heritability to avoid overestimation [78]. This is a valid method, particularly where controls are likely to include misclassified samples even after screening, or screening for multiple phenotypes may lead to super-normal controls which can increase bias particularly when true genetic correlation is low [79]. In the case of ARFID, where onset is frequently in early childhood, screening of controls is likely to improve power when sample size is low, particularly as ARFID is likely underdiagnosed. Utilising existing unscreened control samples in an ARFID GWAS would require careful calculation of the effect of increased sample size over loss of power due to control misclassification, but has the potential to increase control size at little or no added cost.

#### Understanding the genomic architecture of ARFID

Characterising the genomics of ARFID, including its genetic relationships to other disorders, may help clarify biological substrates of aetiology, and inform research questions regarding risk, prevention, outcomes, and interventions. For example, cross disorder analysis in AN, using data derived from GWAS, identified genetic correlations not only with psychiatric disorders, but also with measured physical activity levels and metabolic, lipid, and anthropometric traits, confirming an important metabolic aspect to this disorder [75].

Genetic pleiotropy, and some shared genetic vulnerability between ARFID and other eating disorders or other mental disorders is expected. Substantial genetic overlap was observed in a large study examining five major psychiatric disorders [80], the greatest overlap estimated at 75% of the causal common genetic variants between bipolar disorder and schizophrenia [81]. Similar cross-disorder analyses of ARFID, based on data gained from GWAS, will elucidate how ARFID relates to other psychiatric and neurodevelopmental conditions, and how it relates to, or is distinct from, commonly comorbid medical conditions such as gastroesophageal reflux disease [82]. Methods for examining genetic interrelationships are based on application of linkage disequilibrium score regression (LDSC) [83, 84]. The joint genetic architecture of traits that may correlate with ARFID can be modelled via genomic structural equation modelling, and any causality relationships can then be explored by generalized summary data-based Mendelian randomization [85].

If observed ARFID presentations reflect differing underlying genetic susceptibilities, then a priori we might expect that there would be genetic differences between each ARFID subtype. In addition, it is reasonable to propose that a core set of genetic factors will also be shared by all subtypes. Examination of the genetic overlap of these presentations can be examined by GWAS meta-analysis of ARFID cases split into presentation-specific subgroups, and performing subsequent genetic correlation. The extent of genetic variation between ARFID with and without comorbidity will be of particular interest, and will require GWAS study designs that accrue rich phenotyping data to support such analyses.

Observations at the diagnostic level that limited intake, limited variety, and aversive ARFID presentations may be related to AN psychopathology, neurodevelopmental disorders, and anxiety disorders including OCD respectively, can be tested empirically and such results may impact our understanding of ARFID aetiology. For example, if at the genetic level a dominant aversive ARFID presentation appears to share more loci with OCD than it does with the other ARFID presentations, then this may shed doubt on the diagnostic validity of the current DSM ARFID classification. Similarly, using genetic information to understand if ARFID shares more common aetiology with EDs or with neurodevelopmental disorders will enhance conceptualisation of the disorder, and have potential impacts on management and treatment.

Historically, nosology has developed without knowledge of the profile of underlying heritable factors present in conditions, and has been based primarily on symptomology. There is little doubt that, going forward, GWAS findings will be utilised in genetically-informed nosology which may challenge the DSM paradigm [86]. It is increasingly clear that in the case of psychiatric disorders, traditional symptomatic diagnostic boundaries are less distinct than previously thought [86–89]. For ARFID, if presentation-specific genetic profiles can be established, that are distinct from other comorbid conditions, this will likely inform the diagnostic nomenclature for potential ARFID subtypes, and would serve to either confirm the existing presentations as true subtypes, or help to refine subtype boundaries.

**Fig. 1:**
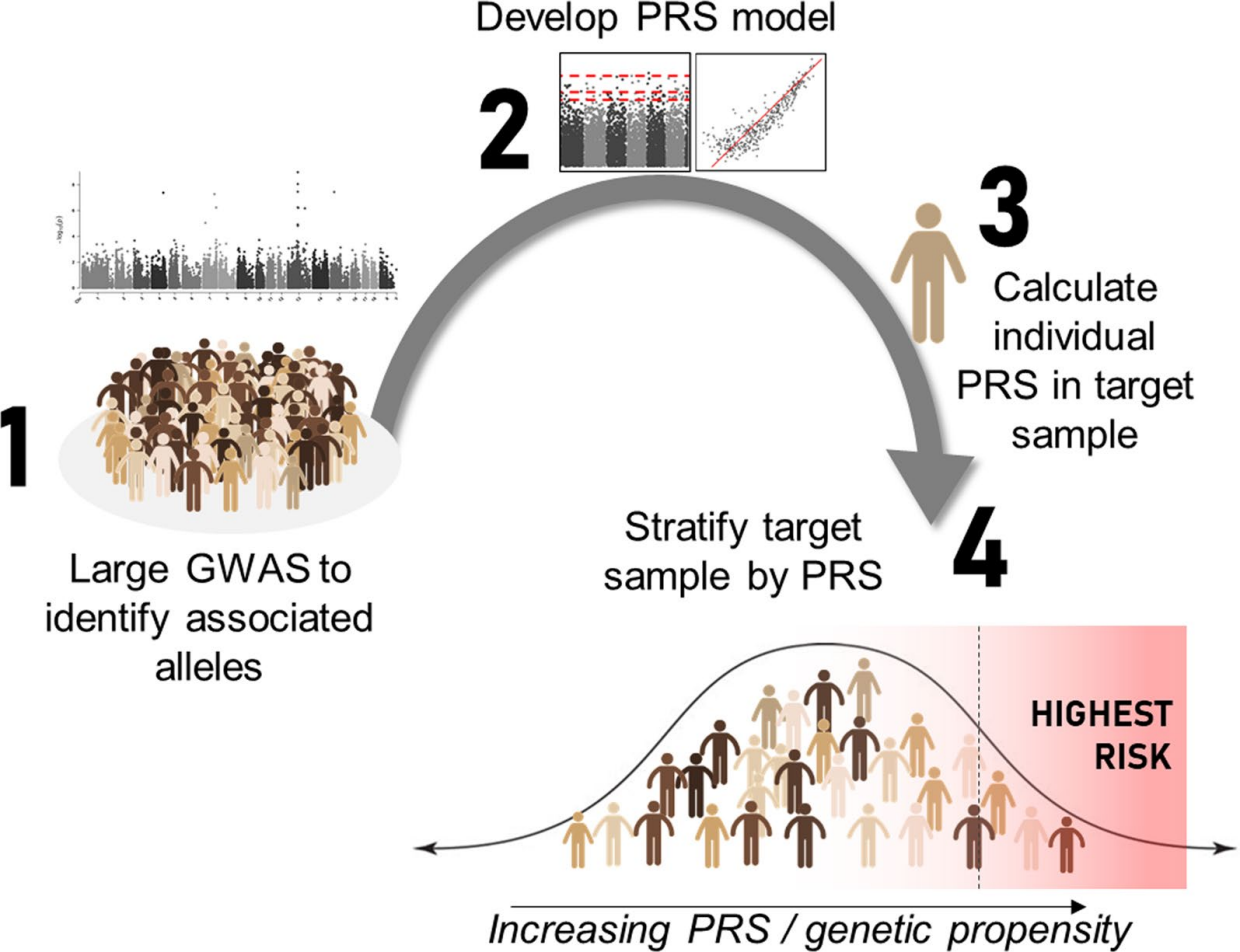
Polygenic risk score (PRS) calculation to identify high risk individuals. 1. Disorder-specific GWAS on largest possible sample to identify associated alleles. 2. Derive a polygenic risk score model from the GWAS data, which incorporates associated SNPs weighted for size of effect. 3. The polygenic risk model can be applied to individuals in a target sample (independent of GWAS sample) to calculate a single polygenic risk score (PRS) that reflects genetic propensity to the phenotype. 4. Identify highest risk individuals based on genetic propensity alone, or combine PRS with information on factors such as environment, family history, and clinical measures to improve predictive ability

At the individual level, leveraging the information provided by a well powered population-level GWAS, information on risk variants (even those of small effect) for ARFID, can be incorporated into a single polygenic risk score (PRS) which is a predictor of an individual’s genetic susceptibility to a particular trait or disorder (Fig. 1). A well performing ARFID PRS analysis could identify risk prior to condition onset, and stratify cases based on genetically informed nosology [90]. PRS analysis can also contribute to the understanding of the genetic architecture of a clinically heterogenous condition in the presence of comorbidity or strongly related traits. As demonstrated in major depressive disorder (MDD), with a well phenotyped GWAS, PRS analysis across multiple comorbid traits identified differential associations with various MDD clinical subtypes [91]. PRS analysis may also answer questions around varying genetic predisposition (or diathesis) between ARFID presentations.

An early application of an ARFID PRS is reported by Koomar et al. [54] to explore correlations with several neuropsychiatric and morphological traits within a cohort of autistic children and their parents. Significant positive associations with metabolic syndrome and neuroticism in parents were found. Appetite (limited intake) and fear (aversive) measures in the probands were associated more with metabolism, whilst the picky measure (limited variety) was more associated with neurodevelopment. It will be important to extend this work in cohorts where ARFID is the primary diagnosis (rather than ASD) as those with ARFID without ASD are likely to exhibit different characteristics.

### Further investigations

The analyses made possible by GWAS may enable a genotypic distinction between children who experience transient developmentally normative fussy eating, threshold ARFID, and enduring ARFID that persists beyond childhood. Do these presentations represent different points on a continuum of fussy eating behaviour susceptibility, or qualitatively different entities? GWAS also provide the starting point for future biological investigations. A few significantly-associated variants identified in GWAS correlate with protein coding changes that infer disease susceptibility, but most are instead involved in regulating other genes [92, 93]. Understanding the genes that are regulated in ARFID will offer insights into the fundamental biological pathways underpinning the disorder. These genes may act in biological systems that provide potential targets for known or novel drugs, providing an opportunity to discover new relevant treatment pathways.

## Discussion

ARFID is a diagnostic category which has been recently established and relatively under-researched. Recent literature suggests that compared to other EDs, ARFID patients are younger at presentation and ARFID may be more prevalent in males (at least in paediatric treatment settings). A presentation-specific classification is also showing some promise, however, there is much that is still uncertain and inconsistencies remain, particularly in understanding the relationship between these presentations, and also between ARFID and comorbid traits or conditions.

A key issue is that these data have been largely drawn from paediatric or age-mixed samples of children, adolescents, and young adults. Data from the adult population are scarce, and few large-scale epidemiological studies with valid measures of ARFID have been performed to ascertain accurate prevalence in the general non-clinical population. As more data are collected, the diagnostic criteria may change and clinical utility may improve. Accumulating evidence on dimensions such as precipitating factors, gender prevalence, age at onset, duration of illness, outcome, and comorbidity will further inform clinical management and treatment development.

Elucidation of the genetic architecture and biological pathways involved in a condition that can have severe and debilitating consequences should be a fundamental aim. It is expected that ARFID, like other EDs, will be moderately heritable. Traits seen as part of the ARFID presentation profile show moderate to high heritability estimates. An ARFID GWAS will elucidate how ARFID relates to other eating disorders, and other psychiatric, neurodevelopmental, and metabolic/anthropometric phenotypes to refine aetiology, and inform nosology.

Recruiting a suitably large sample for an ARFID GWAS is realistically achievable if a collaborative, multinational approach is taken. The Psychiatric Genomics Consortium (PGC) has been instrumental in coordinating study populations large enough to perform highly powered GWAS in many psychiatric disorders including ADHD [94], ASD [95], bipolar disorder [96], major depression [97], schizophrenia [56], Alzheimer’s disease [98], OCD [99], Tourette syndrome [100], post-traumatic stress disorder (PTSD) [101], substance use disorders [102], and EDs [103, 104]. The Eating Disorders Working Group of the PGC has expanded to include ARFID, and identification of existing samples and collection of new samples is beginning. A large, well phenotyped, sample and careful GWAS design is the first step to ensuring that genetic associations discovered are sufficiently specific to ARFID and not driven by other factors.

Consortium science unifies innovative and highly reproducible procedural oversight, with the skills and expertise of researchers and specialists around the world. This global approach enables recruitment of participants from a wide and diverse source, including from localities without the population density to otherwise achieve a GWAS-suitable cohort. To successfully harmonise phenotypic data from multiple study populations, consistent implementation of standardised assessments for ARFID will be critical in the study design. As ARFID can present from infancy to adulthood, age-appropriate assessments will be required such as parent report (for young children), or multi-informant child-parent instruments. Considering the scarcity of published literature on ARFID in adult populations, recruitment of participants from a wide age range will benefit analyses on the lifetime impact of a diagnosis.

A blueprint of how to achieve a large-scale, multinational GWAS cohort has already been provided in the PGC-led Eating Disorder Genetics Initiative (EDGI) [104]. EDGI utilises a standardised set of phenotypic assessments provided as an online survey, with DNA sampling via an at-home saliva sampling kit mailed directly to the participant. Removing barriers to participation, and collecting genetic samples from diverse sources is key to understanding how the disorder occurs in a wide range of populations, and importantly, so as not to perpetuate health disparities [105, 106]. The approach presented in EDGI significantly eases the burden associated with in-person or phone-based interviewing, and ensures the sample collection is both non-invasive and convenient to the participant, and easily scalable for researchers. All that is required to achieve this in ARFID is funding for local research, motivated participants, and researchers and collaborators willing to usher ARFID into the arena of interest.

## Conclusion

Although the body of literature on ARFID is growing since its recognition as an ED in the DSM-5, there is still much that is unknown about epidemiology, clinical characteristics and treatment of this condition and fundamental genetic investigations where ARFID is the primary focus are still absent. Evidence from a preliminary ARFID GWAS (in an autism sample) and heritability estimates of ARFID- related traits, support a role of substantial genetic influence in ARFID. A consortium approach to sample ascertainment, as previously exemplified by the PGC, will be instrumental in delivering a well powered and more comprehensive GWAS for ARFID. This analysis is a vital step to allow investigation into genetic factors that shape the risk, presentation, course of disease, and treatment options of ARFID, as well as enabling important follow-up functional genomic studies. Immediate goals from a genetic analysis of ARFID should be to refine aetiology, and deliver a genetically-informed nosology which will guide future study design and conceptualisation of this debilitating disorder.

## Data Availability

Not applicable.

## Abbreviations

ADHD: Attention deficit hyperactivity disorder
AN: Anorexia nervosa
ARFID: Avoidant restrictive food intake disorder
ASD: Autism spectrum disorder
BED: Binge eating disorder
BMI: Body mass index
BN: Bulimia nervosa
CBT: Cognitive behavioural therapy
CNV: Copy number variant
DSM: Diagnostic and Statistical Manual of Mental Disorders
ED: Eating disorder
EDGI: Eating disorder genetics initiative
GWAS: Genome-wide association study
LDSC: Linkage disequilibrium score regression
OCD: Obsessive compulsive disorder
PANDAS: Paediatric autoimmune neuropsychiatric disorder associated with streptococcal infections
PANS: Paediatric acute-onset neuropsychiatric syndrome
PGC: Psychiatric Genomics Consortium
PRS: Polygenic risk score
PTSD: Post-traumatic stress disorder
SNP: Single nucleotide polymorphism
